# A practical approach to acromegaly management in Latin America

**DOI:** 10.1007/s11102-013-0531-z

**Published:** 2013-11-21

**Authors:** Marcello D. Bronstein, Oscar D. Bruno, Alin Abreu, Ruth Mangupli, Moisés Mercado

**Affiliations:** 1 Neuroendocrine Unit, Division of Endocrinology and Metabolism, Hospital das Clínicas, University of São Paulo Medical School, Av. Dr. Eneas de Carvalho, 255, 7ºandar, sala 7037, São Paulo, CEP 05403-000 Brazil; 2 Division of Endocrinology, Hospital de Clínicas, University of Buenos Aires, Buenos Aires, Argentina; 3 Endocrinología, Centro Médico Imbanaco Cali, Cali, Colombia; 4 Section of Neuroendocrinology, Department of Neurosurgery, Hospital Universitario, Caracas, Venezuela; 5 Faculty of Medicine, Universidad Nacional Autónoma de México, Mexico City, Mexico; 6 Head Endocrine Service, and Experimental Endocrinology Unit, Hospital de Especialidades, Centro Médico Nacional Siglo XXI, Instituto Mexicano del Segero Social, Mexico City, Mexico

**Keywords:** Acromegaly, Latin America, Somatostatin analogs, Dopamine agonists, Pegvisomant, Guidelines

## Abstract

**Introduction:**

Evidence-based treatment guidelines have undoubtedly advanced medical practice and supported optimal management of acromegaly, but their application may be hampered by limited access to the latest treatment options.

**Methods:**

In this retrospective, narrative review, the authors revisited existing treatment guidelines for acromegaly in Latin America. These were considered in conjunction with published evidence chosen at the authors’ discretion.

**Findings:**

In a socially and economically diverse region, such as Latin America, any regional practice guidelines need to appreciate that recommended treatment options, such as surgery by expert pituitary surgical teams and drug therapies, especially somatostatin analogs, are often not available due to limited resources. In these instances, physicians may be obliged to apply less effective therapeutic options.

**Conclusions:**

The current article looks at the practical aspects of acromegaly management in Latin America and discusses this in the context of existing guidelines. Furthermore, we consider potential strategies to make better use of resources through combination and multimodal approaches to treatment.

## Introduction

Diagnosing and managing acromegaly can be a challenge, even in situations where clinicians have access to all the latest diagnostic and treatment modalities (see other articles in this issue [1–4]). This can be complicated further if diagnosis is delayed and/or access to healthcare resources is limited. Failure to identify acromegaly early and to provide optimal disease management often can lead to significant morbidity, severely impaired quality of life and reduced life-expectancy.

Latin America (with between 550 and 600 million people in total) is a region with wide variations in development, poverty, income inequality, literacy, and life expectancy both between and within individual countries [5]. National health care systems in Latin American countries also vary widely in their organizational structure and provision of healthcare services [6]. Access to healthcare resources varies widely not only among countries, but also within countries and even within cities or provinces.

Economic considerations to prioritize resource allocation decisions are increasingly being used in Latin America, but the use and application of formal Health Economic Evaluations or Health Technology Assessments remains suboptimal [6, 7]. Although countries previously relied on technology assessment reports from outside the Latin America region, there is increasing use of region-specific reports, which are considered more relevant [6].

Evidence-based treatment guidelines have undoubtedly advanced medical practice and supported optimal prescribing for acromegaly, but tend to be developed within the context of optimal access to the latest treatment options. Against this background of diversity and disparity in Latin America, any regional practice guidelines for the management of acromegaly need to appreciate that the recommended options may often not be available due to limited resources.

### Epidemiology of acromegaly in Latin America

Although epidemiologic data are relatively limited over the Latin America region as a whole, national acromegaly registries are starting to provide a reliable picture of diagnostic and treatment patterns, as well estimates for the prevalence of the disease [8, 9]. For instance, the Mexican Acromegaly Registry (EpiAcro), which now includes over 1,400 patients, gives an estimated prevalence of 14 cases per million, which is lower than other parts of the world, suggesting under diagnosis [8, 9]. Time between onset and diagnosis ranged from 5 to 17 years, and approximately one-third of patients had an invasive tumor at diagnosis. Pituitary surgery was the most common primary treatment option (73 % of patients), whereas radiosurgery was the primary treatment option in only 3 %. The remainder received pharmacological therapy with somatostatin analogs (SSAs) (15 %) or dopamine agonists (9 %). Approximately 60 % of patients undergoing primary surgery did not achieve biochemical remission and required secondary therapy. For secondary treatment, 36 % had radiosurgery, 36 % received SSAs, 19 % received dopamine agonists and 26 % had surgery. The latest data suggest that 34 % of patients in the registry are in biochemical remission, 40 % have active disease and 27 % are stable on pharmacological therapy.

## Guidelines for the management of acromegaly

Since 2000, the Acromegaly Consensus Group has developed several international guidelines and consensus statements regarding the management of acromegaly [10–14]. The latest major update to these guidelines was published in 2009, based upon evidence available in 2007, and a further consensus on diagnosis and treatment of acromegaly complications was published in 2012 [14, 15]. A meeting held in Mexico City in 2007 led to expert panel recommendations on the management of acromegaly specifically in Latin America, and these were published in 2010 [16]. Guidelines have also been developed at the national level in Mexico and Brazil [17, 18].

**Fig. 1:**
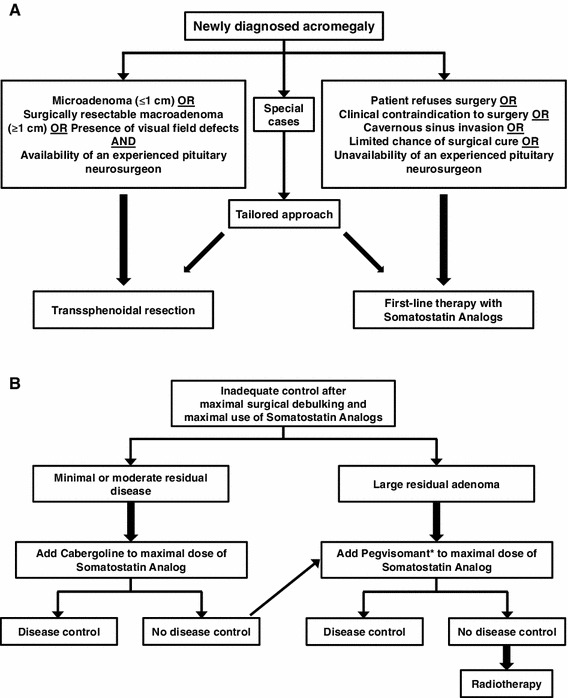
Treatment algorithm for choosing first-line therapy in Latin American patients with newly diagnosed acromegaly (**a**) or those uncontrolled after surgery and SSAs (**b**). *Because pegvisomant is not available in all Latin American countries, radiotherapy might be considered as an additional treatment option for patients not controlled after maximal doses of somatostatin analogs and/or cabergoline. (Reproduced with permission from [16])

All the latest versions of these guidelines and consensus statements generally recommend either surgery or, if there is a low probability of surgical cure, SSAs as primary therapy in acromegaly (see Fig. 1 for the algorithm developed by the Latin American Expert Panel [16]). The SSAs are considered the pharmacological treatment of choice because they fulfill all the requirements for the primary treatment of acromegaly by reducing tumor volume, controlling disease symptoms and achieving biochemical control in the majority of patients. Nevertheless, it should be pointed out that the best results were obtained in patients with mild-to-moderate serum GH elevation. Furthermore, a selection bias could occur in retrospective studies due to the withdrawal of unresponsive patients. Evidence to support the use of SSAs as the primary pharmacological therapeutic option is now extensive and includes several studies involving Latin American populations [19–23]. Furthermore, all the guidelines generally recommend SSA therapy as the next line of treatment in patients with insufficiently controlled GH secretion after surgery.

In the settings above, dopamine agonists (principally cabergoline) are generally reserved for patients with relatively low GH and IGF-I concentrations or those in whom an oral drug is preferred over injectable therapy (as is the case for all SSA formulations). The GH receptor antagonist pegvisomant is generally reserved for third-line therapy. Radiosurgery is considered to be an option in selected cases when no disease control is achieved with surgery and pharmacological therapy, especially if pegvisomant is not available.

While considering the recommendations highlighted above, one key issue raised by the Latin American Panel is that of limited access to resources at the local level [16]. This message was reinforced repeatedly by the Panel, e.g.:

- “Since not all the diagnostic tools and treatment options are available in all Latin American countries, physicians need to adapt their clinical management decisions to the available local resources and therapeutic options”.
- “…treatment of patients with acromegaly in Latin America is influenced by local issues of cost, availability and expertise of pituitary neurosurgeons, which should dictate therapeutic choices.”
- “…a range of management approaches may not be available for many patients with acromegaly and the feasibility and cost should be considered in the implementation of local guidelines.”
- “Access to SSAs is a key issue in Latin America, as treatment is not always subsidized by government agencies.”
- “Physicians in Latin America should tailor appropriate treatments or combinations for each patient based on the clinical presentation and availability of resources”.
  - “…judgment is required to indicate the first-line therapy, taking into account the local experience and availability of resources.”

## Revisiting the expert panel recommendations on the management of acromegaly in Latin America

Little has changed in terms of new therapies in the market since the publication of those recommendations (although there may have been changes in access to individual therapeutic options at the local level). However, guidance was minimal regarding the best approach to take in cases where access to resources is restricted, although it was noted that “Radiotherapy may be indicated in selected cases….when local issues of cost preclude other therapies.” The preceding section summarizes the broad consensus panel efforts regarding recommendations for the management of acromegaly in Latin America. The following sections provide our own suggestions on how to gain the most benefit from available resources within the context of those recommendations. This may assist in providing a more practical, relevant and flexible approach to acromegaly treatment across Latin America.

In view of the recommendations that SSAs generally represent the first-choice pharmacological therapy in acromegaly, it is worth highlighting the way in which this therapy can be optimized and costs reduced [24]. Addition of cabergoline (a less effective, but also less expensive oral agent that is widely available in Latin America) may improve response in patients uncontrolled with SSAs alone, thus improving the cost-effectiveness of these agents [24–27]. Although pegvisomant is expensive, requires daily injections and has only limited availability across Latin America, it can be effective as add-on therapy in partial responders to SSAs and there are several other potential advantages to using this combination in selected patients [24, 28, 29]. For instance pegvisomant/SSA combination therapy may be associated with 1) improved insulin sensitivity and quality of life compared with using SSAs alone, 2) better control of tumor size compared with pegvisomant alone, and 3) reduced pegvisomant doses and thus cost savings [24, 28, 29]. For acromegalic women with mild IGF-1 elevation, estrogens represent an inexpensive alternative to pegvisomant, as they act as a post-receptor noncompetitive GH antagonist by stimulating hepatic socs2; however, the risk of thrombosis should be taken into account [30].

In centers with sufficient surgical expertise, surgical reduction of tumor mass can improve the outcome of SSA treatment in acromegalic patients resistant to primary therapy with SSAs [24, 31]; similarly, SSA treatment may improve surgical outcomes [32]. Nevertheless, access to skilled surgeons should not dictate the choice of therapy, if pharmacological treatment is indicated. In well-controlled patients, it may be possible to increase the interval between doses without losing efficacy [33]. In a small subset of patients, it may also be possible to permanently discontinue SSA therapy, suggesting that these agents might provide permanent beneficial functional changes in GH release (at least in some patients) [34].

Cabergoline may be seen as an alternative (albeit less effective) low-cost pharmacological treatment in situations where SSAs are not available. However, it should be emphasized that this remains a suboptimal first choice and health authorities should be encouraged to improve access to recommended pharmacological therapies (i.e., SSAs). Radiosurgery and modern external beam radiotherapy can be an effective, low-cost and reasonably safe means of controlling acromegalic activity, although it has a long efficacy latency period [35, 36]. However, it should be emphasized that the choice of radiation techniques must be based on tumor characteristics and if possible should be performed using stereotactic devices [37]. Therefore, health authorities should be aware of the cost/benefit ratio of less efficacious therapies, which, albeit leading to potential savings, may be hampered by the social and economic burden of co-morbidities present in uncontrolled acromegalic patients, as well as, in the case of radiotherapy, the costs of full pituitary hormone replacement in the frequent cases of progression to panhypopituitarism.

## Other factors that may contribute to improved care in acromegaly in Latin America

Delayed diagnosis is a problem in many parts of the world, including Latin America [8, 9, 38, 39]. Consequently, many patients present with advanced disease that may not be suitable for surgery, thus limiting treatment options. There is, therefore, a need to increase awareness about acromegaly—this has the potential to increase the chances of early diagnosis and promote early referral to expert centers, thus increasing treatment options, improving long-term outcomes and reducing costs [40]. Education of general practitioners and the adoption of simple screening techniques based on phenotypic alterations may be one cost-effective method for identifying acromegaly early [41]. Medical students, nurses, and the general public may also be good candidates for increased awareness programs.

Implementation of support networks to provide adequate patient follow-up is another important component of acromegaly management. Patients on pharmacological therapy require life-long treatment, but compliance can be poor and many patients will not receive adequate long-term therapy if follow-up support is insufficient [42]. Furthermore, a significant proportion of patients will change their biochemical status during long-term follow-up after surgery and may require a modification of management strategies [43].

Finally, it should be stressed that diagnosis and management of acromegaly is complex and requires the involvement of multidisciplinary expert teams. As such, the comprehensive care, education and support of patients with acromegaly are best carried out in designated Pituitary Centers of Excellence (COE) [44]. Increasing access to COEs across Latin America is thus a key aspect of improving care in the region.

## Conclusions

One of the greatest challenges in providing consensus recommendations regarding the management of acromegaly in Latin America is the diversity of access to treatment and reimbursement policies across this large population of between 550 and 600 million. The wide variations in access to healthcare resources across Latin America, which may manifest in terms of access to surgical and/or pharmacological resources and the availability of adequate patient support networks, make it difficult to provide pan-regional recommendations for the management of acromegaly.

In some countries, the availability and cost of SSAs and other drugs, such as pegvisomant, as well as the availability of surgical expertise, are critical issues. Thus, some key recommendations can only be followed when these resources are available and physicians may be obliged to apply therapeutic options that are not indicated by the international guidelines.

In the absence of access to first line recommended pharmacological therapy (i.e., SSAs), physicians may have to rely on less expensive, less effective drugs (most notably cabergoline) or other less well-tolerated treatment modalities (e.g., radiosurgery). Nevertheless, opportunities exist for more flexible use of first choice therapies, such as SSAs, in order to optimize treatment and reduce costs, although economic outcome data are lacking. There is also a common need across countries to increase awareness about acromegaly—this could improve early diagnosis and lead to improved outcomes and reduced costs.

In conclusion, delayed diagnosis and limited access to healthcare resources can compromise optimal management of patients with acromegaly. This situation may be particularly notable in parts of Latin America due to the economic and social diversity across the region. Although opportunities exist to improve management through optimal use of existing limited healthcare resources, there is a need for increased access to recommended therapies, especially SSAs. Furthermore, the creation of regional acromegaly patient registries, such as EpiAcro, should help to improve understanding of diagnostic and therapeutic trends in different regions of Latin America.
